# Medical Society in Northern Iowa

**Published:** 1859-08

**Authors:** F. Andros, H. C. Martin

**Affiliations:** President; Secretary


### MEDICAL SOCIETY IN NORTHERN IOWA.

### MEDICAL CONVENTION.

Pursuant to a previous call, a large number of the medical practitioners of this and adjoining towns assembled at Jones & Bass' Hall in this city at 1 o'clock p. m., and proceeded to organize a Medical Society.

Amos Warner, of Elkader, was appointed chairman of the meeting, and H. C. Martin appointed Secretary.

Dr. Warner briefly stated the objects of the meeting to be the formation of a society of physicians, thereby securing unanimity of action among practising physicians, and the advancement of true medical science, in this portion of Iowa.

On motion, Dr. Scott, F. Andros and John Low were appointed a committee to examine credentials. This committee soon reported that they had examined as required and found the credentials of Amos Warner, H. C. Martin, A. Blanchard, J. T. H. Scott, F. Andros, John Low, Horace Hamilton, William Hyde and P. G. Parker satisfactory, and entitled to a seat in this body. The report of the committee was received and the committee discharged.

F. Andros, John Low and A. Blanchard were appointed a committee to draft a Constitution and By-Laws.

F. Andros, J. T. H. Scott and John Low were appointed a committee to report a Fee Bill.

On motion of Dr. Andros, Wm. Hoffbauer, of Guttenberg, was admitted to a seat in this convention.

The committee on Constitution and By-Laws handed in their report, which was accepted and the committee discharged. The different Articles of the Constitution were then read by the Secretary,—taken up separately, discussed and adopted, with little alteration of the draft as recommended by the committee.

By Art. 1 of the Constitution, as reported by the committee, the society shall be known by the name of "The North Iowa Medical Society."

The officers required by the Constitution are a President, Vice-President, Secretary, Treasurer and three Censors, to be elected annually by ballot. After the adoption of the Constitution by the convention, it proceeded to the election of permanent officers, with the following result: F. Andros, President; Amos Warner, Vice-President; H. C. Martin, Secretary; Wm. Hoffbauer, Treasurer; J. T. H. Scott, A. Blanchard and P. G. Parker, Censors.

Dr. Andros, on being installed into the office of President of the society, made a few appropriate remarks alluding to the importance of the enterprise, and expressing a great interest in the welfare of this society.

The report of the committee on Fee Bill was accepted and the committee discharged.

Article 19th of the By-Laws elicited a warm discussion, but the Article was finally adopted by an almost unanimous vote. The following is the Article:

The members of this society shall refuse to counsel with any irregular practitioner and all such physicians as shall refuse, after a reasonable time, to recognize the code of this, and be governed by the Fee Bill adopted by this society.

This article was adopted in consideration of the vast number of empirics who, by their disreputable practice, not only injure their patients but destroy the confidence that should exist between the public and the educated physicians.

Four meetings are to be held annually; the anniversary meeting to be held the first Wednesday in June of each year, quarterly meetings to be held the first Wednesdays of September, December and April.

John Low and A. Blanchard were appointed by the President to read papers on some medical subject or subjects at the next meeting.

Some other general business was transacted, when, on motion of Dr. Parker, the society adjourned, to meet at Garnavillo on the first Wednesday of September next.

F. ANDROS, *President.*

H. C. MARTIN, *Secretary.*

